# Space Use of African Wild Dogs in Relation to Other Large Carnivores

**DOI:** 10.1371/journal.pone.0098846

**Published:** 2014-06-04

**Authors:** Angela M. Darnell, Jan A. Graf, Michael J. Somers, Rob Slotow, Micaela Szykman Gunther

**Affiliations:** 1 Department of Wildlife, Humboldt State University, Arcata, California, United States of America; 2 School of Life Sciences, University of KwaZulu-Natal, Durban, South Africa; 3 Centre for Wildlife Management, Centre for Invasion Biology, University of Pretoria, Pretoria, South Africa; 4 Smithsonian Conservation Biology Institute, Front Royal, Virginia, United States of America; Panthera, United States of America

## Abstract

Interaction among species through competition is a principle process structuring ecological communities, affecting behavior, distribution, and ultimately the population dynamics of species. High competition among large African carnivores, associated with extensive diet overlap, manifests in interactions between subordinate African wild dogs (*Lycaon pictus*) and dominant lions (*Panthera leo*) and spotted hyenas (*Crocuta crocuta*). Using locations of large carnivores in Hluhluwe-iMfolozi Park, South Africa, we found different responses from wild dogs to their two main competitors. Wild dogs avoided lions, particularly during denning, through a combination of spatial and temporal avoidance. However, wild dogs did not exhibit spatial or temporal avoidance of spotted hyenas, likely because wild dog pack sizes were large enough to adequately defend their kills. Understanding that larger carnivores affect the movements and space use of other carnivores is important for managing current small and fragmented carnivore populations, especially as reintroductions and translocations are essential tools used for the survival of endangered species, as with African wild dogs.

## Introduction

Large carnivores play a key role in regulating terrestrial ecosystems [1], and competition between them is considered a key ecological factor affecting carnivore species within the same guild [2]. Past studies have focused on carnivore-prey interactions or exploitative competition between carnivores, while recent studies have increasingly recognized the significant effects carnivores can have on each other through interference competition [3]–[5]. Carnivores of the same guild may compete for similar prey resources, often resulting in smaller species either being excluded from, or actively avoiding, areas with higher densities of the larger competitor [6]–[8]. In Nepal, leopards (*Panthera pardus*) avoided habitats where tiger (*Panthera tigris*) densities were high [9], while another study found a significant pattern of avoidance of spotted hyenas (*Crocuta crocuta*) by the smaller brown hyenas (*Hyaena brunnea*; Mills & Mills, 1982). Studies have also suggested that gray wolves (*Canis lupus*) displace and exclude coyotes (*Canis latrans*) from preferred habitat [11]. These studies illustrate the widespread pattern of avoidance and exclusion of smaller carnivores with less competitive advantage due to interference competition. Overall, competition between intraguild carnivores can confine spatial distributions, restrict habitat use, reduce prey encounter rates and food intake, and increase mortality of competitors [6].

Extensive diet overlap between large African carnivores is associated with high levels of competition [5], [12]. This is particularly evident in the interactions between African wild dogs (*Lycaon pictus*), lions (*Panthera leo*), and spotted hyenas (hereafter referred to as hyenas). Wild dogs are consistently found at lower population densities than any other sympatric carnivore [13]. Interference competition from larger carnivores may affect African wild dog movements, provoking active avoidance spatially and (or) temporally in the areas in which they range [14], [15].

African wild dogs, once widespread across sub-Saharan Africa, are now endangered [16]. Reasons suggested for the species' decline, such as habitat fragmentation, persecution by humans, and disease, affect all large carnivores in sub-Saharan Africa, yet wild dogs in particular continue to decline in many areas [6]. Because large carnivores are mostly confined to protected areas, they may be forced to interact more frequently than they might have historically, increasing the effects of interference competition [6]. This is especially true in smaller parks that are significantly separated from other populations, such as occurs in the highly fragmented metapopulation of wild dogs in South Africa [17].

To gain a better understanding of the potential threats to wild dog persistence in South Africa, we utilized location data collected concurrently on large carnivores in Hluhluwe-iMfolozi Park (HiP) in northern KwaZulu-Natal province. We tested the hypothesis that African wild dog space use was affected by other large carnivores. Lions are a more significant threat to wild dogs as they regularly injure and kill them [18]–[20], while hyenas pose a significant, but less serious, threat by stealing wild dog kills [21]. Considering the differing levels of threat, and that lion distributions in HiP are generally clustered and hyenas are more evenly distributed on the landscape [22], [23], we predicted that wild dogs' space use would differ relative to their two main competitors, preferentially avoiding lions more strongly than hyenas. The main goal of this study was to provide information on the spatial dynamics of large carnivores in order to better manage wild dog populations.

## Methods

### Study Area

Hluhluwe-iMfolozi Park is located between 28°00′ and 28°26′S and 31°43′ and 32°09′E in the northern KwaZulu-Natal province, Republic of South Africa. The park is approximately 900 km^2^ and is enclosed by an electrified fence that was begun in the 1940s and finished by the late 1970s. HiP is about 300 km south of Kruger National Park, which contains the closest persisting population of wild dogs. HIP is the second largest protected area and one of the most popular wildlife viewing areas in South Africa, receiving tourists from around the world [24]. Hunting is not allowed in the park, although poaching has been an intermittent problem addressed by management in the form of daily patrols. Human habitations inside the park include a field ranger station in each of the five sections of the park, a tourist lodge and small community for the park's researchers and staff for the Hluhluwe section of the park, a tourist camp and nearby houses for the iMfolozi staff, and seven small tourist bush camps. HiP contains 250 km of roads accessible to the public, including a regularly used, high-speed, tarmac road that bisects the park and 238 km of management roads only accessible to park personnel. The roads inside the park are primarily used by the park's tourists and staff, with the exception of the tarmac road, which is used by the public to cut through the park.

The park is primarily savannah thornveld, with much of the park dominated by shrubland *Acacia* spp. [25], [26]. The landscape contains numerous hills and valleys ranging from 60 m to 590 m above sea level. The subtropical climate of the park has unimodal rainfall peaking in summer from November to February. Average temperatures are warm to hot, ranging from 13–35° C [27]. The heterogeneous environment of HiP supports a large and diverse prey base, from red duiker (*Cephalophus natalensis*) to greater kudu (*Tragelaphus strepsiceros*), and, as a result, a wide variety of both small and large predators, from black-backed jackals (*Canis mesomelas*) to lions. The abundance of lions in HiP was around 100 individuals at the start of this study [28], and the hyena population ranged from 300–400 individuals [22]. Wild dogs in HiP feed largely on nyala (*Tragelaphus angasi*) and impala (*Aepyceros melampus*; Kruger, Lawes & Maddock, 1999).

### Study Species

Wild dogs have distinctive social behaviors that make them a near-obligate cooperative species: not only do they hunt more successfully in packs, but packs must have a minimum number of members to successfully reproduce [30], [31]. Large pack size not only allows wild dogs to hunt more efficiently, but also allows them to prey on species that will be more energetically profitable, as well as enhancing defense of their kills from scavengers [21], [32]. These benefits decrease the quantity of required hunts, which reduces the pack's energetic costs and risk [33], [34], increasing overall fitness [35]. Within a pack, usually only the alpha male and female reproduce, although subordinate males and females may breed on occasion [36], [37]. Breeding occurs once per year, with nearly equal-length periods for denning (pups restricted to den) and post-denning (pups out of den but too young to travel with the pack on hunts), while the remainder of the year the pack is more mobile and traveling together.

The wild dog population in HiP is a product of several reintroductions beginning in 1980–1981 [38], [39], and translocations to and from HiP continued up to the time of the study [18]. As packs have established home ranges in all sections of the park over the years since reintroduction (KwaZulu-Natal Wild Dog Advisory Group), it is not likely that these translocations significantly bias the space use of wild dogs in HiP. In the past, this population has undergone large fluctuations and was extremely susceptible to stochastic demographic or environmental events [39], making additional information about the population extremely valuable for conservation decisions [18].

### Ethics Statement

All research was approved by the provincial wildlife organization in South Africa (Ezemvelo KwaZulu-Natal Wildlife) and the Institutional Animal Care and Use Committees (IACUC) of the Smithsonian National Zoo and Humboldt State University.

### Data Collection

The large carnivores of HiP were monitored regularly from January 2002 through December 2004. Individual wild dogs (at least 2 per pack in 6 packs) and lions (at least 1 per pride in 12 prides) were radio-collared (Sirtrack, Inc., New Zealand; African Wildlife Tracking, South Africa) by HiP management staff. Additionally, all wild dog den sites were located each year. During every year of the study, at least one member of every pack and pride was collared. We monitored locations, movements and behaviors of all wild dog packs and lion prides on a daily or weekly basis using the VHF collars. Monitoring times ranged from middle of the night to middle of the day but focused on peak hours of carnivore activity (the hours just before and after sunrise and sunset). Although this may have created a temporal bias in our data, our primary objective was to examine interactions during the times when the carnivores were most active and not while they were inactive and resting during the day. Thus, focusing on peak activity hours was most informative for our study goals. Visual sightings of all large carnivores were also recorded on an opportunistic basis (although the majority of the data for wild dogs and lions was collected by telemetry; only a handful of these data points were opportunistic sightings and likely would not have had a significant impact on the results). Date, time and GPS location of all animals observed were recorded for all visual large carnivore sightings (both tracked and opportunistic sightings), as were number of animals observed, and their age, sex and behavior. When visual sightings were not possible, triangulation data were used (3.5% of the data, 108 out of 3,113 locations).

One hyena was collared in October 2004 and monitored until December 2004. Additionally, our data include 11 hyena call-ups (a technique used for estimating population numbers of hyenas and not designed to attract hyenas from outside their native home ranges; see Graf et al. 2009 for details): 5 days in October 2003 (at 20 different locations) and 6 days in August and September 2004 (at 24 different locations).

We used GPS coordinates of all independent points (different pack, pride or clan sub-group separated by >12 hours) to create maps using ArcGIS (v 10.0, Environmental Systems Research Institute, Redlands, California) to determine the spatial habits of carnivores. These data were separated by year (as conditions such as precipitation that may affect space use can be variable year to year), and because wild dogs exhibit distinct behavioral changes throughout the year [40], also into three periods of equal length: denning (May-Aug), post-denning (Sept-Dec), and non-denning (Jan-Apr). Although, there may be some small overlap (several days), these seasons correspond to fairly consistent changes in the behavior and range of wild dogs. Data for all analyses were separated by pack or pride as these are fairly cohesive groups [36], [41]. While members of these groups may not be together 100% of the time, the analyses used in this study required that individual units be independent, and the movements of members of the same pack or pride are not independent of one another.

Hyenas, however, have much different social systems than wild dogs and lions. They live in permanent, territorial social groups called clans [42]. Clans are fission-fusion societies that contain several subgroups, and individuals often change subgroups [43]. Thus, as most hyenas were not known individuals assigned to a particular clan, the majority of hyena data are opportunistic sightings of individuals and groups that could not be allocated to specific subgroups. Of the 5 seasons in which we had enough data points to complete analyses, 1 included the collared hyena, 3 included call-up data, and 2 contained only opportunistic sightings. Statistical tests were considered significant at alpha of 0.05 [44].

### Static Interactions

We assessed the static interactions (spatial interactions without a temporal aspect) among carnivore species using home ranges and core use areas, when at least 50 locations within a season were available [45]–[47]. Home ranges and core use areas were determined for each wild dog, lion and hyena group using a bivariate normal fixed-kernel estimator in Geospatial Modeling Environment (v 0.5; H.L. Beyer, Spatial Ecology, LLC) with smoothing factors calculated using a diagonal plug-in in R statistical software (v 2.14.1; R Foundation for Statistical Computing, Vienna, Austria). From the kernel density layer, we used Geospatial Modeling Environment to obtain isopleth polygons: 95% for home range and 50% for core use areas.

When the home range and core use areas overlapped, we determined the mean percentage of overlap as: *mean overlap*  =  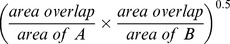 [48]. We used 2-sample t-tests to test for differences between percentage of overlap of home ranges and core use areas between species and ANOVA to test for differences between seasons.

We also determined 3-dimensional overlap in space use which takes into account a third dimension: intensity of use in an area [46]. We used the kernel density raster layers, which reflected peaks of use within a home range, to obtain a volume of intersection: 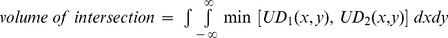
where UD_1_ and UD_2_ are the utilization distributions (the kernel density layers) for each species [46], [49]. The volume of intersection (3-dimensional overlap) measures the degree of overlap in shape and location of two utilization distributions. This index ranges from no overlap (0) to complete overlap (1). We used 2-sample t-tests and ANOVA to test for differences between species and seasons for 2- and 3- dimensional overlap in home range and core use areas. For non-overlapping core use areas, we determined the average distance separating each species using centroids (the central, most heavily used point) of each core use area, for neighboring groups.

### Dynamic Interactions

When there was any overlap between carnivore home ranges, we analyzed dynamic spatial and temporal interactions. In contrast to the previous static interactions, dynamic interaction analyses incorporated the temporal aspect of the association between the species. Based on guidelines from Kernohan et al. [46], we calculated the distance between simultaneous locations (defined as <12 hours) of two groups and compared the distances to what would be expected at random. We calculated observed distances (D_O_) as:
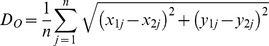
where for *n* pairs of locations for each group, x_1_and y_1_ and x_2_ and y_2_ (for all occasions of *j*) are the UTM coordinates for species 1 and 2, respectively. In other words, we took the reciprocal of the summed Euclidean distances between the two groups. The expected distances (D_E_) for all recorded observations (for all occasions of *j* and *k*) were calculated as: 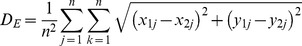



We combined all interactions for each species group and compared differences between observed (D_O_) and expected distances (D_E_) using a Wilcoxon signed-rank test [44], [50]. If there was a statistically significant difference between the observed and expected distances, we concluded that the species were expressing either attraction or avoidance.

We used methods recommended by Minta [48] to further analyze spatial and temporal interactions between carnivore species. We tested the null hypothesis, that for each group of species, α and β, one species moved randomly, using the overlap area independent of the other [48]. We tested this hypothesis when the two species groups had any overlap in home range or core use area and where there were at least 30 independent points for each group within that overlap area. Locations for each group that had overlapping home ranges or core use areas within a season (den, post-den, non-den) were placed into one of the following categories: (1) both groups of species were absent from the overlapped area (n_11_), (2) only species group α was present in the overlapped area (n_21_), (3) only species group β was present in the overlapped area (n_12_), or (4) both groups of species were present in the overlapped area (n_22_). Expected frequencies of presence and absence in overlap areas were calculated using areas as recommended by Minta [48]. We then totaled the observed frequencies of presence and absence for each group and determined the expected frequencies using the proportion of overlapped area between the two species in relation to total home range area.

## Results

### Static Interactions

There were 1,647 independent wild dog pack locations for three packs, 1,466 independent lion pride locations for 12 prides, and 428 independent hyena locations. There were sufficient locations (at least 50 within a season) to analyze space use within nine seasons between wild dogs and lions and five seasons between wild dogs and hyenas. The average number of locations (± SE) used to create home ranges for each group was: wild dog packs: 111.8±20.8, lion prides: 189.1±45.1, and hyena sub-groups: 70.0±8.3. The number of locations used to determine home range was not significantly correlated with the size of the home range (wild dogs: r_22_ = 0.231, *p* = 0.550; lions: r_23_ = −0.036, *p* = 0.926; hyenas: r_3_ = 0.407, *p* = 0.496).

**Figure 1:**
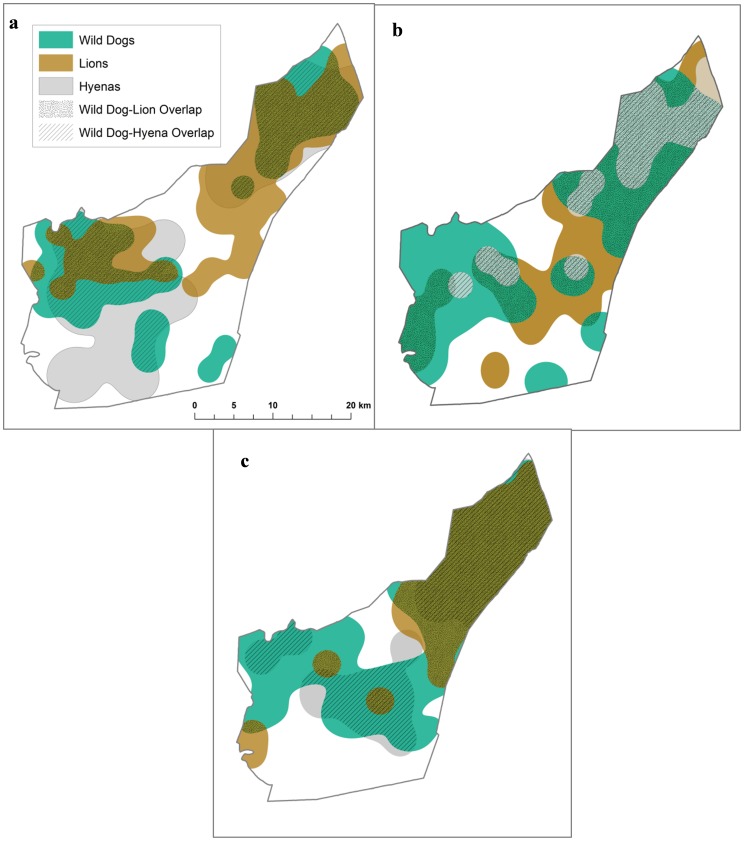
Home ranges of African wild dogs, lions, and spotted hyenas during the (a) denning period, (b) post-denning period, and (c) non-denning period in 2004 in Hluhluwe-iMfolozi Park, South Africa.

Home ranges of wild dogs, lions, and hyenas varied greatly throughout the study period and between seasons (Figure 1). Throughout the study period wild dog home ranges varied between 33.50–200.98 km^2^, lion home ranges between 71.87–170.41 km^2^, and hyenas between 33.54–99.53 km^2^. Wild dog home ranges were not significantly different from either lions or hyenas (*p* = 0.622 and *p* = 0.263, respectively), however lion home ranges were significantly larger than hyena ranges (t_10_ = −2.363, *p* = 0.039).

When core use areas did not overlap, wild dog packs remained an average of 16.6±2.1 km (n = 19) away from neighboring lion prides during the denning season, whereas packs only maintained an average distance of 6.7±1.6 km (n = 10) from lion prides during the other times of year (collectively ‘not denning’), (t_26_ = 3.76, *p* = 0.001). In contrast, the average distance from wild dogs to hyenas during the denning season (2.0±1.9 km, n = 2) was not significantly different from ‘not denning’ (2.5±6.0 km, n = 6; *p* = 0.894).

**Figure 2:**
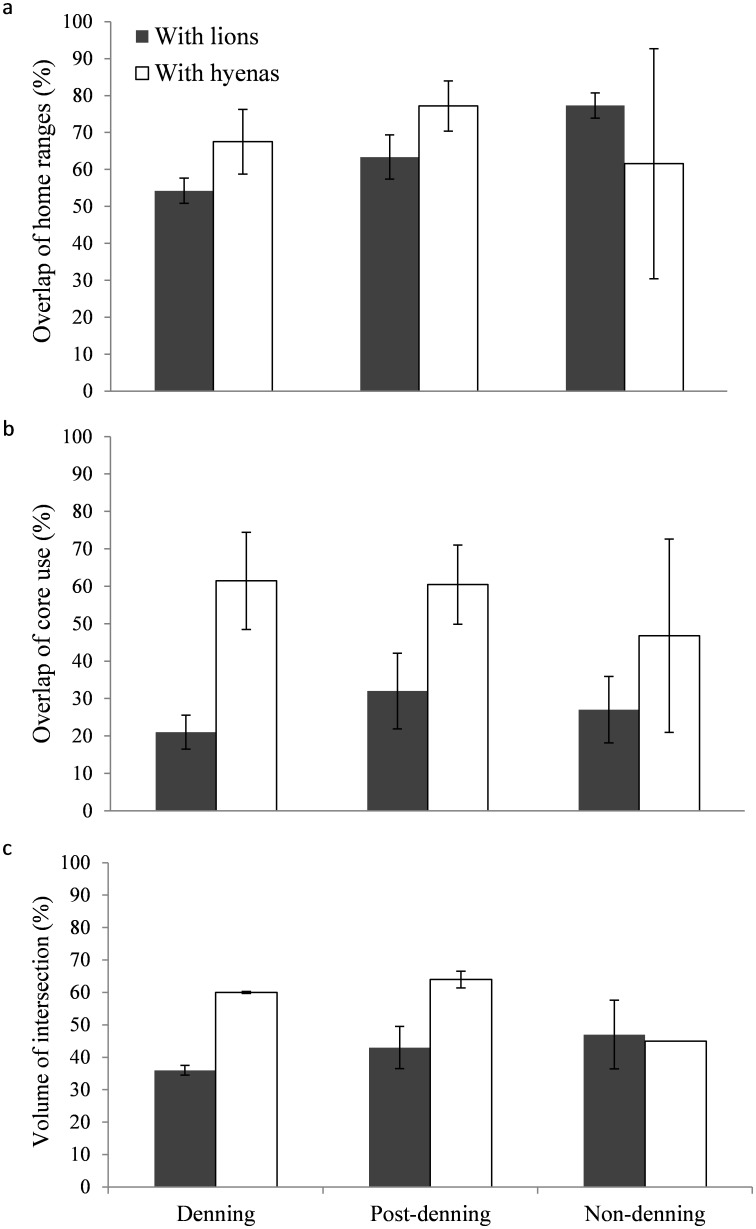
Mean percentage overlap (± SE) of (a) home ranges, (b) core use areas and (c) and volume of intersection (3-dimensional overlap) of home ranges of wild dogs with lions (n = 9) and spotted hyenas (n = 5) between periods in Hluhluwe-iMfolozi Park, South Africa, 2002–2004.

Overlap in home ranges was significantly lower during denning than non-denning season for wild dogs and lions (F_17_ = 6.85, *p* = 0.008; Figures 1, 2a), but not for wild dogs and hyenas (*p* = 0.887). Overlap in core use areas did not differ significantly between seasons for any species (*p* = 0.635 with lions, *p* = 0.745 with hyenas; Figure 2b). Overlap in core use areas was significantly less than overlap in home ranges for wild dogs and lions (t_18_ = −7.86, *p*<0.001), but there was no significant difference for wild dogs and hyenas (*p* = 0.052). Core use areas of wild dogs overlapped significantly more with hyenas than with lions (t_7_ = −3.34, *p* = 0.016; Figures 2b).

When taking into account intensity of use in an area, the volume of intersection of home ranges (3-dimensional overlap) between wild dogs and lions was significantly less than the mean (2-dimensional) overlap overall (t_8_ = −4.96, *p* = 0.001), as well as within each season: denning (t_6_ = 4.89, *p* = 0.001), post-denning (t_5_ = 2.31, *p* = 0.035), and non-denning (t_2_ = 3.03, *p* = 0.047; Figure 2c). However, there was no significant difference for wild dogs and hyenas overall (*p* = 0.223) or between seasons (*p* = 0.96, 0.75, and 0.80 for denning, post-denning, and non-denning, respectively). There was no significant difference between (3-dimensional) use of home ranges and core areas of wild dogs and lions (*p* = 0.69). However, the overlap for core use areas of wild dogs and hyenas was significantly higher than for home ranges (t_3_ = −3.95, *p* = 0.029). Finally, there was significantly less 3-dimensional overlap of wild dogs with lions during the denning season compared to ‘not denning’ (post-denning and non-denning seasons combined) (t_5_ = −2.26, *p* = 0.037), but this pattern did not hold with hyenas (*p* = 0.99).

### Dynamic Interactions

When we included a temporal aspect to the interaction analyses between species, we found that wild dogs were significantly further from lions than expected when comparing denning to not denning seasons (t_7_ = 2.04, *p* = 0.04), supporting the previous static interaction analyses. Wild dogs were significantly closer to hyenas than expected overall (W_s_<0.001, *p* = 0.011, n = 7), but there was no significant difference between seasons (*p* = 0.55).

### Interactions in overlap areas

The analysis of interactions of wild dogs and lions within overlap areas (Appendix A in Appendices S1) indicated, that within home ranges, wild dogs exhibited spatial avoidance of the overlap area 78% of the time; 22% of the time there was (non-significant) random use by wild dogs. Lions demonstrated spatial attraction to the overlap area 67% of the time, with 22% random use and one occurrence of avoidance. Wild dogs exhibited spatial avoidance of overlapping core use areas 12% of the time, spatial attraction 38%. Lions showed 75% attraction to the overlap area and 25% random use. All of the temporal interaction analyses were negative, indicating that solitary use was greater than simultaneous use. For home range overlap, we found 56% spatial avoidance and 44% temporal avoidance of lions by wild dogs. In core use areas, we found 12% spatial avoidance and 88% temporal avoidance of lions by wild dogs in the overlap area.

The analysis of interactions between wild dogs and hyenas within overlap areas (Appendix B in Appendices S1) indicated that within home ranges, wild dogs exhibited spatial attraction to the overlap areas 60% of the time, with 20% non-significant random use and one occurrence of spatial avoidance. Hyenas demonstrated spatial avoidance of wild dogs 80% of the time, with one occurrence of spatial attraction. Within core use areas, wild dogs exhibited 60% spatial attraction and 40% non-significant random use, while hyenas showed 100% spatial avoidance of wild dogs. We found 80% of all interaction analyses significant and of these all were negative (solitary use > simultaneous use). Within both home range and core use areas, hyenas exhibited 75% spatial avoidance of the overlap area and 25% temporal avoidance.

## Discussion

Our results support the hypothesis that African wild dogs utilize space differently relative to their two main competitors in Hluhluwe-iMfolozi Park. Overall, wild dogs remained further from lions than from hyenas, and their core use areas and 3-dimensional space use overlapped significantly more with hyenas than with lions. These results are consistent with Webster et al. [51] who found that wild dogs actively avoid lions more than they avoid hyenas. In addition, other studies have shown that cheetahs (*Acinonyx jubatus*) adjust their behavior more often in response to lions than to hyenas [52], [53].

Competition with its two main competitors, lions and hyenas, can be a major hindrance to wild dog populations [54]. One of the main causes of natural mortality in wild dogs is intraguild predation, most often by lions [19], [20]. Therefore, it is not surprising that wild dog densities are consistently low in areas where lion densities are high [41], [55]. The considerable overlap (49–82%) of home ranges between wild dogs and lions in HiP is likely due to the relatively small size of the park as well as the fact that the park is fenced, resulting in relatively high carnivore densities in HiP [22], [28]. However, as this study suggests, it appears possible for wild dogs to avoid lions through adjustments in core space use [15]. Wild dogs spent a majority of their time in areas free of lions, as was evidenced by the significantly lower overlap of core use areas as compared to home ranges. Additionally, the analysis of 3-dimensional spatial overlap indicated that peaks of space use in the home ranges of wild dogs differed significantly from lions when compared to mean (2-dimensional) overlap. Wild dogs of Pilanesberg National Park, South Africa responded to lions in a similar manner, avoiding areas where lion use was high [15].

Wild dogs remained significantly further from lions during the denning season as indicated from both static and dynamic spatial interaction analyses. It is likely that the packs adjusted their behavior while denning to avoid lions [15], as almost half of all juvenile wild dog mortality is as a result of lion predation [56]. It may be that wild dogs choose den sites far from lion pride core areas, as core use areas (in which all den sites were located) were significantly further from lions during denning seasons. Wild dogs demonstrated temporal avoidance when spatial avoidance was not possible during the denning season, which is likely due to the restricted movement of wild dogs during this time as packs must return to the den after each hunting foray to feed the alpha female and her pups [36]. In our study, home ranges contracted 33–76% during denning seasons, and core use areas were 16–289% larger when the wild dogs were not denning (post and non-denning seasons). During the non-denning season, wild dogs did not alter their space use as much, as both their mean overlap, and overlap incorporating intensity of use, with lions were similar to their overlap with hyenas.

Instances of wild dog attraction to overlap areas, where lion densities are high, likely corresponded to areas of high prey density [57]. Lions have been shown to be distributed according to habitat and landscape characteristics [58] as well as prey availability [14], [59], factors that are often correlated [60]. Furthermore, Hopcraft et al. [61] suggested that it was not simply prey density but prey's susceptibility to capture (related to habitat) that defined fine scale movements of lion prides. Consequently it may be necessary for wild dogs to spend time in those areas to increase their prey encounter rates [41] despite the presence of lions, especially when traveling with growing pups. Thus, wild dogs in HiP exhibited a hierarchal response to lions, primarily utilizing spatial avoidance and secondarily utilizing temporal avoidance when spatial avoidance was not possible (i.e., due to young pups or prey densities).

Wild dogs in HiP did not appear to alter their space use significantly in relation to hyenas. Past studies suggest that hyenas have a negative impact on wild dogs, due to frequent stealing of wild dog kills, a phenomenon termed kleptoparasitism, a largely one-way interaction [21], [34], [55]. A study by van der Meer et al. [62] found that wild dogs selected habitats based on kleptoparasitism risk, avoiding areas with high densities of hyenas. Additionally, daily activity data presented by Saleni et al. [63] showed that wild dogs in HiP are primarily active during periods of low hyena activity, suggesting that wild dogs in HiP temporally avoid interacting with hyenas at this time scale. Other studies, however, have found little effect of hyenas on wild dogs [51], [64], [65]. The space use results of this study provided no evidence of wild dogs avoiding hyenas either spatially or temporally. This pattern is likely even stronger considering that hyena observations were limited and restricted to mostly opportunistic sightings, and there were likely many more hyenas present in areas with wild dogs than were reported. Although this result could be a product of the limited number of collared hyenas, this is unlikely as only 2.6% of the data points (11 out of 428 hyena sightings) used involved simultaneous observation of hyenas and wild dogs.

The lack of avoidance of hyenas by wild dogs is likely due to the relatively large size of wild dog packs in HiP, which can adequately defend their kills against kleptoparasitic hyenas. The average wild dog pack size in the park during the study years was 17 individuals, higher than the average pack sizes in other parks [41]. Whateley and Brooks [66] found the average hyena clan size in HiP to be relatively small: between 9–14 individuals. Since then, hyena numbers have increased in the park through increasing numbers of clans [22], while feeding groups remain small. Large wild dog packs can better defend their kills, and for longer periods of time, than can smaller packs [67]. Kleptoparasitism will only negatively affect wild dogs when hyenas take over kills quickly, as wild dogs can fill their stomachs on a kill within minutes [21], [67]. As it appears that wild dog pack numbers in HiP are large enough to avoid most cases of kleptoparasitism, extra effort to avoid hyenas becomes unnecessary, and in some cases would be detrimental due to the high total energy needs of the packs [34]. Furthermore, this pattern could be indirectly caused by both wild dogs' and hyenas' avoidance of lions, as lions are also a significant source of hyena mortality [42],[68]. It is also possible that wild dogs do not have the opportunity to avoid hyenas (except possibly at finer scales that were undetectable within our study) as their times of activity can overlap [69], [70] and because hyena densities in HiP are relatively high [22].

It has been suggested that conservation of high densities of competing carnivores in small, fenced reserves may not be feasible and may lead to the extinction of the smaller competitor [55], [57]. However, it appears that the wild dog packs of HiP have been able to adapt to life in a small fenced reserve with lions through a combination of spatial and temporal avoidance, adjusting their behavior as necessary based on the life-history stage of the pack (i.e., when denning). It should be noted, however, that the wild dog population in HiP, as in all other reserves in KwaZulu-Natal, is actively managed [71], and this management likely also contributes to the wild dogs' persistence. As the wild dog population in HiP is currently relatively stable (∼100 individuals in 8 packs, KwaZulu-Natal Wild Dog Advisory Group, unpublished data), this study confirms the findings of other recent studies in the South African wild dog meta-population [17], [18], [35], that smaller wild dogs can coexist with larger lions and hyenas in relatively small, fenced reserves, however this may require active management of the wild dog population. Heterogeneity in vegetation and other habitat characteristics likely promotes this coexistence [65], [72]–[76]; unfortunately this study was not able to look at such relationships as it was beyond the scope of our data. We suggest that combining spatial use data with habitat information, as well as data on prey density, would be an important area for future research.

Temporal changes in the population densities of lions and spotted hyenas in relation to wild dogs suggest the limiting effects these dominant competitors have on the density of wild dogs. Wildlife managers and conservationists often simply consider exploitative competition when developing management strategies. As the success of conservation efforts may rely on the interactive role between species, managers should account for competitive relationships between sympatric carnivores when devising management tactics [7], [54], [77]. Our study suggests that taking into account interference competition between species may be equally important. In KwaZulu-Natal province, where most wild dog packs exist in fenced reserves and individual numbers are generally low and often not self-sustaining, management through reintroductions and relocations is common and is part of the provincial conservation strategy [78]. Even in HiP, where the population is relatively stable at this time (2013), introductions are necessary to avoid inbreeding and genetic drift [37], [79]. Thus, the information from our study will be useful, particularly in regards to choosing appropriate reintroduction and relocation sites. Although wild dogs can persist in areas with high densities of larger competitors, when considering reintroduction and translocation sites, it would be better to place new packs in areas with low lion density, as suggested by this study and others demonstrating high wild dog mortality due to lions [18]–[20].

## Supporting Information

Appendices S1 **This file contains Appendix A and Appendix B.** Appendix A. Interaction within overlapping home ranges and core use areas for African wild dogs (A) and lions (B), showing spatial attraction or avoidance by each and interaction between groups (ixn) as well as deviation of odds from random in Hluhluwe-iMfolozi Park, South Africa, 2002–2004. Appendix B. Interaction within overlapping home ranges and core use areas for African wild dogs (A) and spotted hyenas (B), showing spatial attraction or avoidance by each and interaction between groups (ixn) as well as deviation of odds from random in Hluhluwe-iMfolozi Park, South Africa, 2003–2004.
(DOCX)
