# Supplementary material for: Space Use of African Wild Dogs in Relation to Other Large Carnivores
Source: PLoS One. 2014 Jun 4;9(6):e98846. doi: 10.1371/journal.pone.0098846 (PMC4045926; doi:10.1371/journal.pone.0098846)
Supplement: Appendices S1 — This file contains Appendix A and Appendix B. Appendix A. Interaction within overlapping home ranges and core use areas for African wild dogs (A) and lions (B), showing spatial attraction or avoidance by each and interaction between groups (ixn) as well as deviation of odds from random in Hluhluwe-iMfolozi Park, South Africa, 2002–2004. Appendix B. Interaction within overlapping home ranges and core use areas for African wild dogs (A) and spotted hyenas (B), showing spatial attraction or avoidance by each and interaction between groups (ixn) as well as deviation of odds from random in Hluhluwe-iMfolozi Park, South Africa, 2003–2004. (DOCX) [file pone.0098846.s001.docx]

Appendix A.

a)

b)

a)

c)

c)

|  |  |  | Spatial Effects | | | | Interaction Effects | | Odds for each cell^d^ | | | |
| --- | --- | --- | --- | --- | --- | --- | --- | --- | --- | --- | --- | --- |
| Year | Season |  | L_A:Ā_ ^a^ | p_A_^b^ | $L_{B:\bar{B}}$ ^a^ | p_B_^b^ | L_ixn_^c^ | p_ixn_^b^ | n_11_ | n_12_ | n_21_ | n_22_ |
| 2002 | Denning | Home range | -0.699 | 0.001 | 0.852 | < 0.001 | -1.000 | < 0.001 | 0.567 | 1.870 | 0.438 | 0.699 |
|  |  | Core use | -1.103 | 0.002 | 1.877 | < 0.001 | -1.519 | < 0.001 | 0.613 | 4.480 | 0.414 | 0.458 |
|  | Post-den | Home range | -0.687 | 0.002 | 0.230 | 0.314 | -1.579 | < 0.001 | 0.341 | 2.078 | 1.256 | 0.693 |
|  |  | Core use | 2.205 | 0.700 | 4.675 | < 0.001 | -4.755 | < 0.001 | 0.000 | 17.857 | 1.520 | 0.167 |
|  | Non-den | Home range | -1.639 | < 0.001 | 1.044 | < 0.001 | -2.711 | < 0.001 | 0.336 | 9.754 | 0.709 | 0.528 |
| 2003 | Denning | Home range | -0.402 | 0.004 | -0.102 | 0.470 | -1.332 | < 0.001 | 0.386 | 2.012 | 1.345 | 0.500 |
|  |  | Core use | 1.446 | < 0.001 | 1.483 | < 0.001 | -3.363 | < 0.001 | 0.000 | 3.878 | 3.053 | 0.240 |
|  | Post-den | Home range | -0.198 | 0.343 | 0.445 | 0.047 | -0.909 | < 0.001 | 0.734 | 1.586 | 1.146 | 0.367 |
|  |  | Core use | 0.322 | 0.185 | 0.919 | 0.004 | -1.120 | < 0.001 | 0.847 | 1.616 | 1.680 | 0.228 |
|  | Non-den | Home range | -1.069 | < 0.001 | 0.284 | < 0.001 | -0.599 | < 0.001 | 0.169 | 1.746 | 4.837 | 3.448 |
|  |  | Core use | 0.760 | 0.008 | 0.864 | 0.003 | -1.832 | < 0.001 | 0.615 | 2.016 | 2.129 | 0.048 |
| 2004 | Denning | Home range | -0.074 | 0.717 | 1.052 | < 0.001 | -1.278 | < 0.001 | 0.827 | 2.442 | 1.060 | 0.149 |
|  |  | Core use | 0.934 | 0.134 | 2.197 | < 0.001 | -4.483 | < 0.001 | 0.000 | 5.569 | 2.246 | 0.088 |
|  | Post-den | Home range | -2.355 | < 0.001 | 1.190 | < 0.001 | -1.956 | < 0.001 | 0.619 | 1.079 | 5.035 | 0.245 |
|  |  | Core use | 0.530 | 0.050 | -0.474 | 0.092 | -1.796 | < 0.001 | 0.471 | 1.035 | 3.484 | 0.279 |
|  | Non-den | Home range | -0.939 | 0.032 | -0.628 | 0.001 | -1.557 | < 0.001 | 0.339 | 1.683 | 2.053 | 0.448 |
|  |  | Core use | 0.129 | 0.672 | 0.487 | 0.113 | -1.272 | < 0.001 | 0.718 | 1.857 | 1.346 | 0.179 |

^a^ Spatial main effects of A (wild dogs) or B (lions) indicating attraction (+) or avoidance (-) of overlap area

^b^ P-value for χ^2^ test with values < 0.05 indicating effects were significant

^c^ Interaction effects

^d^ Odds indicate departure from expectation of each cell (n_11,_ n_12,_ n_21_, n_22_), with values close to 1 indicating use of shared area

was as expected at random, values < 1 indicating use less than expected, and > 1 indicating use more than expected

Appendix B.

|  |  |  | Spatial Effects | | | | Interaction Effects | | Odds for each cell^d^ | | | |
| --- | --- | --- | --- | --- | --- | --- | --- | --- | --- | --- | --- | --- |
| Year | Season |  | L_A:Ā_ ^a^ | p_A_^b^ | $L_{B:\bar{B}}$^a^ | p_B_^b^ | L_ixn_^c^ | p_ixn_^b^ | n_11_ | n_12_ | n_21_ | n_22_ |
| 2003 | Denning | Home range | 0.979 | < 0.001 | -1.345 | < 0.001 | -1.192 | < 0.001 | 0.591 | 0.520 | 3.283 | 0.564 |
|  |  | Core use | 0.799 | 0.001 | -0.921 | < 0.001 | -0.744 | 0.001 | 0.678 | 0.544 | 2.186 | 0.620 |
|  | Post-den | Home range | 0.014 | 0.956 | -2.097 | < 0.001 | -0.111 | 0.371 | 0.479 | 0.533 | 3.741 | 4.184 |
|  |  | Core use | 0.322 | 0.159 | -1.726 | < 0.001 | -0.523 | 0.002 | 0.568 | 0.568 | 4.217 | 2.269 |
| 2004 | Denning | Home range | -0.558 | 0.011 | -2.054 | < 0.001 | -0.506 | 0.004 | 0.223 | 1.273 | 4.313 | 2.530 |
|  |  | Core use | 1.611 | < 0.001 | -2.764 | < 0.001 | -1.814 | < 0.001 | 0.363 | 0.271 | 10.256 | 1.352 |
|  | Post-den | Home range | 0.590 | 0.018 | -2.446 | < 0.001 | -1.162 | < 0.001 | 0.533 | 0.516 | 1.342 | 2.224 |
|  |  | Core use | 0.393 | 0.163 | -0.738 | 0.008 | -0.919 | 0.001 | 0.645 | 0.848 | 2.857 | 0.833 |
|  | Non-den | Home range | 1.234 | < 0.001 | 0.975 | < 0.001 | -0.764 | < 0.001 | 1.745 | 1.733 | 2.081 | 0.032 |
|  |  | Core use | 2.178 | < 0.001 | -1.668 | < 0.001 | -1.730 | < 0.001 | 1.096 | 0.614 | 0.290 | 9.357 |

^a^ Spatial main effects of A (wild dogs) or B (hyenas) indicating attraction (+) or avoidance (-) of overlap area

^b^ P-value for χ^2^ test with values < 0.05 indicating effects were significant

^c^ Interaction effects

^d^ Odds indicate departure from expectation of each cell (n_11,_ n_12,_ n_21_, n_22_), with values close to 1 indicating use of shared area

was as expected at random, values < 1 indicating use less than expected, and > 1 indicating use more than expected
